# Pathogenesis of Stevens-Johnson Syndrome/Toxic Epidermal Necrolysis With Severe Ocular Complications

**DOI:** 10.3389/fmed.2021.651247

**Published:** 2021-11-17

**Authors:** Mayumi Ueta

**Affiliations:** Department of Ophthalmology, Kyoto Prefectural University of Medicine, Kyoto, Japan

**Keywords:** Stevens-Johnson syndrome (SJS), toxic epidermal necrolysis (TEN), severe ocular complications (SOC), cold medicine, *TLR3*, EP3, *IKZF1*

## Abstract

Stevens-Johnson syndrome (SJS)/toxic epidermal necrolysis (TEN) is an acute inflammatory vesiculobullous reaction of the mucosa of the ocular surface, oral cavity, and genitals, and of the skin. Severe ocular complications (SOC) are observed in about half of SJS/TEN patients diagnosed by dermatologists and in burn units. Ophthalmologists treat SOC, and they tend to encounter the patients not only in the acute stage, but also in the chronic stage. Our investigation of the pathogenesis of SJS/TEN with SOC led us to suspect that abnormal innate mucosal immunity contributes to the ocular surface inflammation seen in SJS/TEN with SOC. We confirmed that cold medicines such as NSAIDs and multi-ingredient cold medications are the main causative drugs for SJS/TEN with SOC. Single nucleotide polymorphism (SNP) association analysis of cold medicine-related SJS/TEN with SOC showed that the Toll-like receptor 3 (*TLR3*)-, the prostaglandin-E receptor 3 (*PTGER3*)-, and the *IKZF1* gene were significantly associated with SNPs and that these genes could regulate mucocutaneous inflammation including that of the ocular surface. We also examined the tear cytokines of SJS/TEN with SOC in the chronic stage and found that IL-8, IL-6, IFN-γ, RANTES, eotaxin, and MIP-1β were significantly upregulated in SJS/TEN with SOC in the chronic stage. Only IP-10 was significantly downregulated in SJS/TEN with SOC in the chronic stage. This mini-review summarizes the pathological mechanisms that we identified as underlying the development of SJS/TEN with SOC.

## Introduction

Stevens-Johnson syndrome (SJS) is an acute inflammatory vesiculobullous reaction of the skin, the mucosa of the ocular surface, the oral cavity, and of the genitals; its severe phenotype is called toxic epidermal necrolysis (TEN).

Ophthalmologists tend to encounter the patients in the chronic stage, they can find it difficult to differentiate between SJS and TEN because the vesiculobullous skin lesions present in the acute stage have healed, so they tend to report SJS and TEN with severe ocular complications (SOC) broadly as “ophthalmic SJS” (1).

Approximately half of all SJS/TEN patients diagnosed by dermatologists and in burn units presented with SOC, e.g., severe conjunctivitis with pseudomembrane and ocular surface epithelial defects in the acute stage (2). When ophthalmologists encounter patients in the chronic stage, based on a confirmed history of acute-onset high fever, serious mucocutaneous illness with skin eruptions, and involvement of at least two mucosal sites including the ocular surface and ocular sequelae, e.g., severe dry eye, symblepharon, trichiasis, conjunctival inversion to the cornea, their diagnosis tends to be SJS/TEN (1, 3–6). SJS/TEN patients with severe conjunctivitis, epithelial defects, and pseudomembrane on the ocular surface in the acute stage often suffer serious ocular sequelae such as severe dry eye and vision disturbance that affect their daily life (7).

We reported cold medicines, including multi-ingredient cold medications and NSAIDs, as the main causative drugs of SJS/TEN with SOC (1, 4–6, 8, 9). About 80% of the SJS/TEN with SOC patients treated at the Kyoto Prefectural University of Medicine developed SJS/TEN within several days after taking medicines to combat the common cold (1, 4–6, 8–11).

We also observed that patients with SJS/TEN with SOC presented with opportunistic infection of the ocular surface by bacteria, especially MRSA and MRSE. The MRSA and MRSE detection rate was higher on the ocular surface of patients with SJS/TEN with SOC than in individuals with other devastating ocular surface disorders (12). SJS/TEN with SOC patients presented with persistent inflammation of the ocular surface even in the chronic stage. Their ocular surface inflammation was exacerbated by colonization with MRSA and MRSE, although, under normal conditions, colonization with these bacteria need not elicit ocular surface inflammation (1). Based on these observations we considered the possibility of an association between a disordered mucosal innate immune response and SJS/TEN with SOC. We postulated that a balance between the mucosal innate immunity of the ocular surface and bacterial pathogenicity is important. When host mucosal innate immunity is normal, commensal bacteria are in a symbiotic relationship with the host, however, when the host mucosal innate immunity is compromised, commensal bacteria including MRSA and MRSE can become pathogenic and contribute to the ocular surface inflammation seen in SJS/TEN with SOC (1).

We have been investigating the pathogenesis of ophthalmic SJS for more than 10 years. This mini-review summarizes our research findings on the pathological mechanisms underlying SJS/TEN with SOC.

## Genes Associated With SJS/TEN With SOC and Their Functions

Although SJS/TEN with SOC can be induced by drugs, not all patients taking these drugs develop SJS/TEN with SOC. Since the incidence of SJS with SOC is very low, we suspected a genetic predisposition (1, 3) and first performed single nucleotide polymorphism (SNP) association analysis using candidate genes associated with innate immunity. We also carried out genome-wide association studies (GWAS) and found several susceptibility genes for SJS/TEN with SOC. Thereupon we subjected some of these susceptibility genes to function analysis using mouse models.

### TLR3

*TLR3* recognizes double-stranded (ds) RNA, a component of the life-cycle of most viruses and mimics polyI:C. Among toll-like receptors (TLRs) *TLR1*—*TLR10, TLR3* is expressed most intensely on the ocular surface epithelium. Its expression there is stronger than in mononuclear cells and we documented that *TLR3* was able to induce many cytokines and genes on the ocular surface (13–15). SNP association analysis of *TLR3* revealed that in Japan, *TLR3* SNPs were significantly associated with SJS/TEN with SOC (8, 16–18).

Using a murine model of experimental allergic conjunctivitis as a model for ocular surface inflammation, we examined *TLR3* gene function in *TLR3* knock-out (KO)- and *TLR3* transgenic (*TLR3*Tg) mice. We found that ocular surface inflammation was significantly reduced in *TLR3* KO- and significantly increased in *TLR3*Tg mice (19). We also reported that *TLR3* was expressed in the epidermis of the skin and that in a murine model of contact dermatitis, the severity of skin inflammation was significantly lower in *TLR3* KO mice and significantly greater in *TLR3*Tg mice than in wild-type mice (20). Yasuike et al. (21) made the same findings in a murine atopic dermatitis model as we did using a murine model of ocular surface inflammation and contact dermatitis. These findings led us to suspect that *TLR3* was able to positively regulate mucocutaneous inflammation of the skin and ocular surface (22) and might contribute the mucocutaneous inflammation seen in patients with SJS/TEN with SOC (22).

### EP3

GWAS and additional analysis revealed that the prostaglandin E receptor 3 (*PTGER3*) gene was significantly associated with CM-SJS/TEN with SOC in Japan and Korea (4, 17, 23).

We performed function analysis of the *PTGER3* gene whose protein is EP3, one of four receptors (EP1, EP2, EP3, and EP4) of prostaglandin E_2_. Cold medicine ingredients, e.g., acetaminophen and NSAIDs, e.g., ibuprofen and loxoprofen, suppress the production of prostanoids including PGE_2_ (1, 8, 22). PGE_2_ acts on EP3 in the ocular surface epithelium and epidermis, and negatively regulates ocular surface- and skin inflammation (24, 25). Kunikata et al. (26) reported that EP3 negatively regulates respiratory tract inflammation. We suggested that the suppression of PGE_2_ production by cold medicines might contribute to the pathogenesis and onset of CM-SJS/TEN with SOC (1, 4, 6, 8, 11, 22) because PGE_2_ acts on EP3 and negatively regulates mucocutaneous inflammation (24–26).

Our examination of EP3 protein expression on the human ocular surface showed that EP3 protein levels were much lower in the conjunctival epithelium of patients with SJS/TEN with SOC than in the controls, i.e., patients with conjunctival chalasis or chemical burns (27). We postulated that EP3 expression might be strongly down-regulated on the ocular surface of patients with SJS/TEN with SOC and contribute to ocular surface inflammation in these patients (27).

### IKZF1

Using the Affymetrix AXIOM genome-wide ASI 1 array we performed GWAS of samples from 117 Japanese patients with CM-SJS/TEN with SOC and 691 controls (28). The *IKZF1* gene was strongly associated with CM-SJS/TEN with SOC (6) and our meta-analysis of samples from Japanese-, Korean-, Indian-, and Brazilian patients showed a significant genome-wide association between CM-SJS/TEN with SOC and *IKZF1* [rs4917014 (G vs. T), odds ratio (OR) = 0.5, *p* = 8.5 × 10^−11^] (6), suggesting that *IKZF1* may be a universal marker for susceptibility to this disease (6).

Function analysis of *IKZF1* SNPs revealed that the ratio of the splicing isoforms *Ik2/Ik1* may be affected by these SNPs, which are significantly associated with susceptibility to CM-SJS/TEN with SOC and that the function of Ikaros, the protein of *IKZF1*, might be enhanced in CM-SJS/TEN with SOC (6). Ikaros, a transcription factor that regulates numerous biological events, has been reported to regulate important cell-fate decisions involved in the development of adaptive immunity (29).

We suspected that the epithelium played a role in the pathobiology of CM-SJS/TEN with SOC (1) because *TLR3* was strongly expressed in ocular surface epithelial cells (13, 14) and keratinocytes (20), and it regulated ocular surface inflammation (19) and dermatitis (20, 21), and because EP3, which negatively regulates mucocutaneous inflammation, was dominantly expressed on the ocular surface epithelium (24), epidermis (25), and the airway epithelium (26).

**Figure 1:**
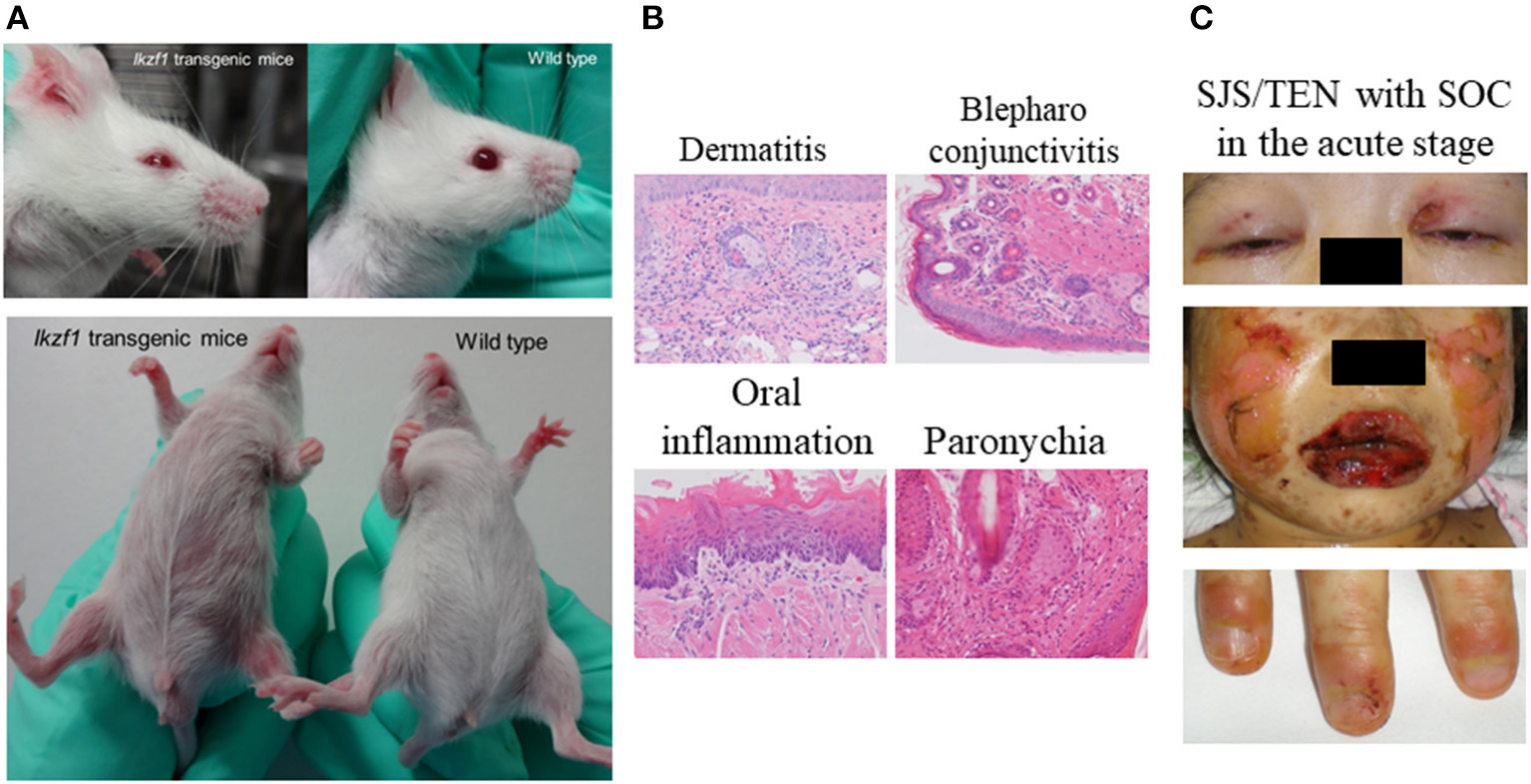
Finding of K5-*Ikzf1*-EGFP transgenic mice (*Ikzf1*Tg). *Ikzf1*Tg mice were introduced the Ik1 isoform into cells expressing keratin 5, which is expressed in epithelial tissues such as the epidermis and conjunctiva. *Ikzf1*Tg mice developed dermatitis and some developed blepharoconjunctivitis **(A)**. Histological analysis showed not only dermatitis but also tissue inflammation of their tongues, blepharoconjunctiva, and paronychia **(B)** as did patients with SJS/TEN with SOC in the acute stage of the disease **(C)**. Reprinted with permission from Ueta et al. (10, 28).

To address this issue we produced K5-*Ikzf1*-EGFP transgenic mice (*Ikzf1*Tg) by introducing the Ik1 isoform into cells expressing keratin 5, which is expressed in epithelial tissues such as the epidermis and conjunctiva. We found that mucocutaneous inflammation was exacerbated in *Ikzf1*Tg mice; they developed dermatitis and some developed blepharoconjunctivitis. Histological analysis showed not only dermatitis but also tissue inflammation of their tongues, blepharoconjunctiva, and paronychia (30) as did patients with SJS/TEN with SOC in the acute stage of the disease (1) (Figure 1).

As our findings suggested that *IKZF1* plays a critical role in maintaining mucocutaneous homeostasis, we proposed that the gene participates in the exacerbation of the mucocutaneous inflammation seen in patients with CM-SJS/TEN with SOC (11, 22, 30).

### Gene–Gene Interactions

Considering the contrasting roles of *Ptger3* and *TLR3* in mucocutaneous inflammation, we looked for an unknown functional interaction between EP3, the protein of *Ptger3*, and *TLR3*. We found that EP3 negatively regulated *TLR3*-dependent ocular surface inflammation (8, 11, 17, 22). Ocular surface inflammation in *TLR3*/*Ptger3*-double-KO mice was decreased to a level similar to that in *TLR3*-KO mice and significantly lower than in wild-type mice (17). Moreover, in conjunctival epithelial cells the EP3 agonist suppressed the production and mRNA expression of polyI:C-induced various cytokines such as RANTES, IP-10 (31), and MCP-1 (32), and TSLP (33).

**Figure 2:**
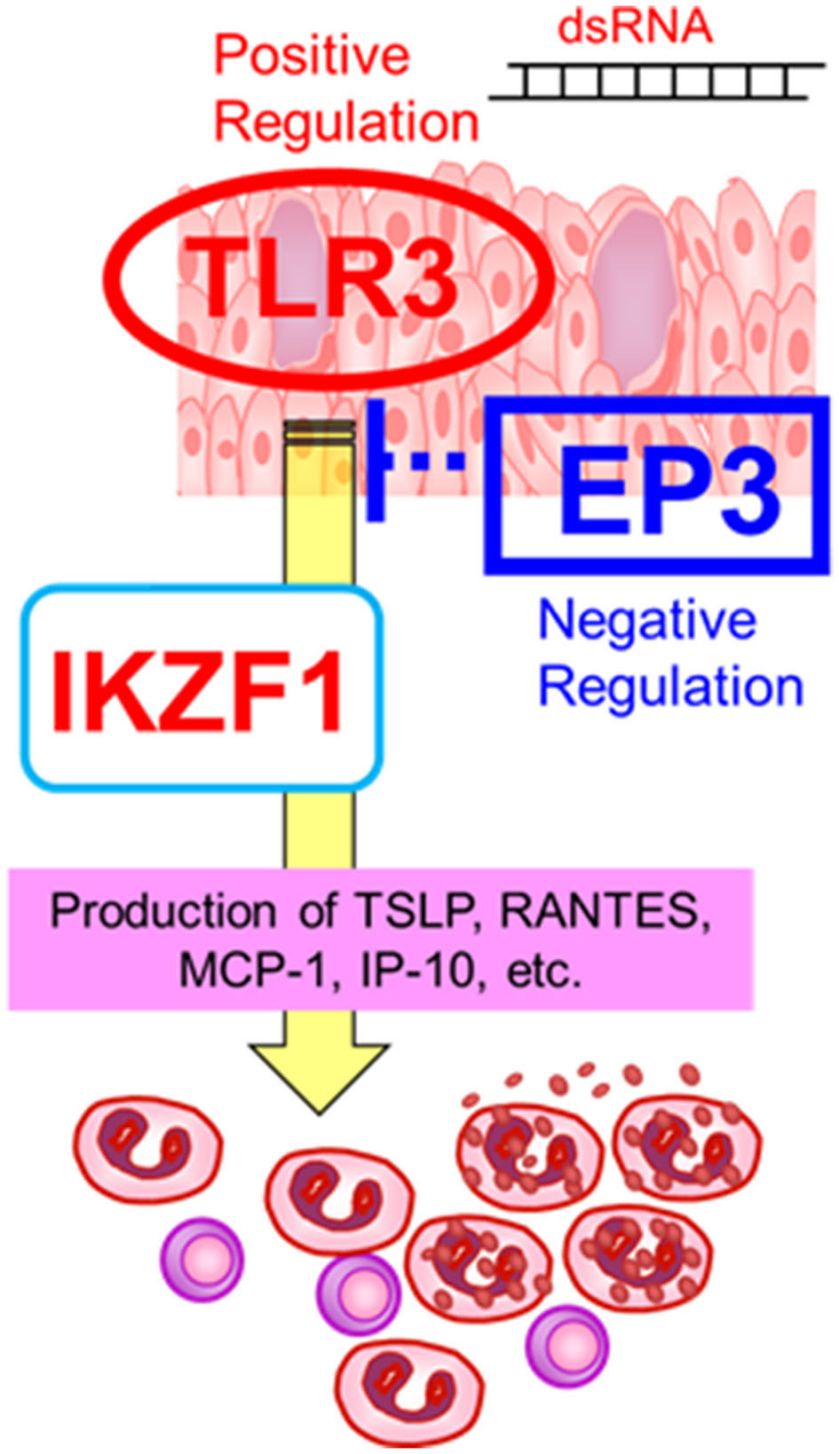
Gene–gene interactions. The susceptibility genes such as *TLR3, PTGER3*, and *IKZF1*, might contribute the mucocutaneous inflammation of SJS/TEN with SOC. Reprinted with permission from Ueta et al. (19).

On the other hand, the expression of *IKZF1* mRNA was upregulated by *TLR3* in human epidermal keratinocytes and conjunctival epithelial cells (30), suggesting an interaction between *TLR3* and *IKZF1* (30). Furthermore, since CM-SJS/TEN with SOC developed in individuals who had taken cold medications to combat the common cold due to viral or mycoplasma, we suspect that not only cold medicines and susceptibility genes such as *TLR3, PTGER3*, and *IKZF1*, but also some microbial infections with, for example viruses or mycoplasma, are important and necessary to trigger the onset of SJS/TEN with SOC (1, 8, 11) (Figure 2).

## Other Studies

We also examined tear cytokines of SJS/TEN with SOC in the chronic stage. IL-8, IL-6, IFN-γ, RANTES, eotaxin, and MIP-1β were significantly upregulated in SJS/TEN with SOC in the chronic stage, while only interferon-γ-inducible protein 10 (IP-10) was significantly downregulated (34). In human corneal and conjunctival epithelial cells, IP-10 is highly induced by the *TLR3* ligand polyI:C (15), possibly as a consequence of abnormal innate immunity that involves the presence of *TLR3* in SJS/TEN with SOC (1, 8, 11).

Moreover, we found that in SJS/TEN patients with SOC, IL-8 was significantly upregulated in eyes with conjunctivalization, neovascularization, or opacification (35). Granzyme B (GrzB) was upregulated in eyes with keratinization, IL-1α in eyes with opacification, and IP-10 was downregulated in eyes with conjunctivalization or neovascularization (all: *p* < 0.05) (35). These observations suggest that IL-8 and IP-10 are involved in conjunctivalization and neovascularization, and that GrzB is involved in keratinization (35).

## Discussion

This mini review suggests that SJS/TEN with SOC is pathogenetically related with a disordered innate immune response.

We identified *TLR3, PTGER3*, and *IKZF1* as susceptibility genes for SJS/TEN with SOC, demonstrated that they are able to regulate mucocutaneous inflammation, including ocular surface inflammation, and reported functional interactions between *TLR3* and *PTGER3*, or *TLR3* and *IKZF1*.

Since CM-SJS/TEN with SOC is a rare and probably has a complex genetic background, it is reasonable to posit multiplicative gene interactions. Multiple susceptibility genes for CM-SJS/TEN with SOC, including innate immunity-related genes such as *TLR3*, may also be involved in functional networks. The absence of a balance between these genes results in abnormal innate immunity and may trigger the development of mucocutaneous inflammation seen in patients with CM-SJS/TEN with SOC (1, 8, 11).

We reported that 80% of our SJS/TEN with SOC patients developed SJS/TEN within several days after taking cold medicines including multi-ingredient cold medications and non-steroidal anti-inflammatory drugs (NSAIDs) to combat the common cold (1, 4–6), suggesting that cold medicines are major causative drugs for SJS/TEN with SOC. We have also suggested that the onset of CM-SJS/TEN with SOC was associated not only with certain drugs but also with putative microbial infection (1, 8, 11, 22).

Moreover, we also analyzed the possible association between human leukocyte antigen (HLA) genotypes and cold medicine-related SJS/TEN (CM-SJS/TEN) with SOC, and found that in the Japanese it was strongly associated with HLA-A^*^02:06 and significantly associated with HLA-B^*^44:03 (5). Interestingly, these *HLA* genotypes were not involved in CM-SJS/TEN without SOC (5), suggesting that the genetic predisposition such as the *HLA* genotype might be different in SJS/TEN patients with/without SOC (5). We also found that CM-SJS/TEN with SOC was significantly associated with HLA-B^*^44:03 in Indian- and Brazilian-, especially Caucasian Brazilian patients, and HLA-A^*^02:06 was associated with CM-SJS/TEN with SOC in Koreans (36).

Based on the totality of the above-cited observations we suggest that in addition to microbial infections and cold medicines, the combination of multiple gene polymorphisms and their interactions might result in abnormal innate immunity and contribute strongly to the onset of CM-SJS/TEN with SOC (1, 8, 11).

We looked for susceptibility genes for SJS/TEN with SOC in the human genome, and investigated their function in a mouse model of ocular surface inflammation and dermatitis. We demonstrated that *TLR3, PTGER3*, and *IKZF1*, susceptibility genes for SJS/TEN with SOC, were able to regulate mucocutaneous inflammation (4, 6, 17, 19–21, 23, 30). Using human samples, we found that EP3 protein levels were much lower in the conjunctival epithelium of patients with SJS/TEN with SOC than in our control subjects (27), and that IP-10, which is greatly induced by the TLR3 ligand on the ocular surface epithelium, was significantly downregulated in the tears of patients with SJS/TEN in the chronic stage (34). These findings on human subjects support our hypothesis that abnormal mucosal innate immunity contributes to the ocular surface inflammation of SJS/TEN with SOC patients (1, 8, 11). Additional studies that focus on the innate immunity of the ocular surface are needed to elucidate the pathogenesis of SJS/TEN with SOC.

## Author Contributions

MU wrote this mini review article.

## Funding

This work was supported by grants-in-aid from the Ministry of Education, Culture, Sports, Science, and Technology of the Japanese government, by the JSPS Core-to-Core Program, A. Advanced Research Networks, and partly supported by grants-in-aid for scientific research from the Japanese Ministry of Health, Labor, and Welfare.

## Conflict of Interest

The author declares that the research was conducted in the absence of any commercial or financial relationships that could be construed as a potential conflict of interest.

## Publisher's Note

All claims expressed in this article are solely those of the authors and do not necessarily represent those of their affiliated organizations, or those of the publisher, the editors and the reviewers. Any product that may be evaluated in this article, or claim that may be made by its manufacturer, is not guaranteed or endorsed by the publisher.
